# Selective loss of microvesicles is a major issue of the differential centrifugation isolation protocols

**DOI:** 10.1038/s41598-021-83241-w

**Published:** 2021-02-11

**Authors:** Annamaria Nigro, Annamaria Finardi, Marzia M. Ferraro, Daniela E. Manno, Angelo Quattrini, Roberto Furlan, Alessandro Romano

**Affiliations:** 1 Division of Neuroscience, Institute of Experimental Neurology, San Raffaele Scientific Institute, Via Olgettina, 60, 20132 Milan, Italy; 2 Department of Biological and Environmental Sciences and Technologies, University of Salento, Lecce, Italy; 3 Department of Mathematics and Physics “E. De Giorgi”, University of Salento, Lecce, Italy

**Keywords:** Biological techniques, Cell biology

## Abstract

Microvesicles (MVs) are large extracellular vesicles differing in size, cargo and composition that share a common mechanism of release from the cells through the direct outward budding of the plasma membrane. They are involved in a variety of physiological and pathological conditions and represent promising biomarkers for diseases. MV heterogeneity together with the lack of specific markers had strongly hampered the development of effective methods for MV isolation and differential centrifugation remains the most used method to purify MVs. In this study, we analysed the capacity of the differential centrifugation method to isolate MVs from cell-conditioned medium using flow cytometry and TEM/AFM microscopy. We found that the loss of MVs (general population and/or specific subpopulations) represents a major and underestimate drawback of the differential centrifugation protocol. We demonstrate that the choice of the appropriate rotor type (fixed-angle *vs* swinging-bucket) and the implementation of an additional washing procedure to the first low-speed centrifugation step of the protocol allow to overcome this problem increasing the total amount of isolated vesicles and avoiding the selective loss of MV subpopulations. These parameters/procedures should be routinely employed into optimized differential centrifugation protocols to ensure isolation of the high-quantity/quality MVs for the downstream analysis/applications.

## Introduction

Extracellular vesicles (EVs) are sub-cellular particles surrounded by a lipid membrane bilayer that are released into the extracellular space from various cell types. They are involved in many physiological/pathological processes and play a major role as potential biomarkers^1–6^. To date, two main groups of EVs have been described, exosomes, small extracellular vesicles (sEVs) of 30–100 nm in diameter originating from the endosomal compartment (multivesicular bodies, MVBs) and microvesicles (MVs), large extracellular vesicles (lEVs) of 100–1000 nm in diameter generated by shedding of the plasma membrane^7–11^. In particular, MVs represent a heterogeneous class of vesicles grouping together different sub-populations which vary in size, composition and molecular cargo^12, 13^. This heterogeneity together with the fact that MV phenotype/diversity remains largely unknown makes their isolation and purification a challenge for researchers.

Currently, a standardized/consensus protocol for MV isolation is still lacking^14–17^ and differential centrifugation (low-speed centrifugation to remove cells/debris followed by high-speed centrifugation to pellet MVs) remains the most commonly used method to purify MVs^18, 19^. However, differential centrifugation presents several limitations including low recovery yield and contaminations by cell debris, organelles, exosomes and/or proteins. Moreover, the use of different parameters such as centrifugation speed, centrifugation time and rotor type can affect yield and quality of sample preparation and cause/explain the high inter-laboratory variability observed in MV yield, purity and enrichment^20–22^. Recently, several studies have systematically explored performances and limits of differential ultracentrifugation as method for isolating/purifying exosomes from cell culture medium and body fluids^23–28^. On the other hand, the actual ability of differential centrifugation protocols to specifically isolate MVs have been largely uninvestigated.

In this study, the differential centrifugation method was carefully tested for its capability to isolate and purify MV populations from cell culture supernatants. By using a flow cytometry approach and TEM/AFM microscopy we found that the quantity and quality of the isolated MVs were strongly affected by parameters (i.e. utilization of swinging-bucket or fixed-angle rotors) and procedures (i.e., implementation of an additional re-washing of P1 fraction) applied to the first (preliminary) low-speed centrifugation step of the differential centrifugation protocol. These results highlight the need to evaluate and define parameters and conditions of all the steps of the differential centrifugation protocol (first step included) in order to ensure isolation of the high-quality/quantity MVs for the downstream analysis and applications.

## Materials and methods

### Cell lines

Human CHME-5 microglial cell lines were cultured in DMEM (Dulbecco’s Modified Eagle’s Medium) supplemented with 10% (v/v) Fetal Bovine Serum (FBS), 1% penicillin–streptomycin and 2 mM l-glutamine (Gibco, ThermoFisher) at 37 °C in a 5% CO2 humidified incubator. Human THP-1 monocytic leukemia cells were cultured in RPMI-1640 medium (Gibco, ThermoFisher) containing 10% (v/v) FBS, 1% penicillin–streptomycin and 2 mM l-glutamine at 37 °C in a humidified atmosphere of 5% CO_2_. THP-1 cells were treated with 100 nM of phorbol myristate acetate (PMA, Sigma-Aldrich Co., St, Louis, MO, USA) for 48 h to induce differentiation of the cells into macrophages.

### MV and exosome isolation

Cells were grown to 70–80% confluency, washed once with phosphate buffered saline (PBS) and growth medium was replaced with similar volume of MV collection medium consisting of PBS with or without extracellular ATP (Sigma-Aldrich) at 1 mM. Cell-conditioned medium was collected after 30 min of incubation at 37 °C and centrifuged for 10 min at 300×*g* at 4 °C using swinging-bucket or fixed-angle rotors to pellet and remove floating cells and cell debris. The supernatant was then centrifuged for 30 min at 10,000×*g* at 4 °C to collect MVs and the pellet of MVs was resuspended in a large volume of PBS and re-centrifuge at 10,000×*g* for 30 min (4 °C) to wash the MV sample. Finally, the resulting supernatant was ultracentrifuged at 110,000×*g* for 90 min at 4 °C to isolate the exosomes. To allow the collection of MVs possibly entrapped with floating cells and cell debris the P1 pellet obtained from the first centrifugation step was subjected to a washing procedure followed by a second MV isolation. P1 pellet was washed with cold PBS and centrifuged at 300×*g* for 10 min. The resulting supernatant was subjected to the high-speed centrifugation step (10,000×*g* for 30 min) to isolate a second MV fraction (MV2). The rotor types/properties and the centrifugation conditions employed during the different steps of the isolation protocol are detailed in Table 1. MV and exosome pellets were resuspended in PBS and all downstream analysis was performed on fresh resuspended samples. For each MV preparation, cells were harvested with trypsin solution, counted using trypan blue staining and the final MV numbers were normalized to the cell numbers.

**Table 1:** Centrifugation and rotor information for MV and exosome isolation.

Centrifugation step	Rotor type	Rotor name	Rotor properties				Centrifugation condition		
			R_min_ (mm)	R_max_ (mm)	Angle (°)	L_sed_ (mm)	RCF_avg_ (g)	RPM	Time (min)
1st step	SW	A-4-62 (Eppendorf)	51	178	–	127	300	1500	10
	FA	F-34-6-38 (Eppendorf)	38.2	107	34	68.8	300	1900	10
2nd step	FA	JA-25.50 (Beckman)	38.5	108	34	69.5	10,000	11,000	30
3rd step	SW	SW 41 Ti (Beckman)	67.4	153.1	–	85.7	110,000	29,800	90

### Flow cytometry analysis

Flow cytometry analysis was performed using the Accuri C6 Cytometer (Becton Dickinson, Franklin Lakes, New Jersey) and data was analysed with FlowJo software (version 10, Tree Star, Ash-land, OR).

Fresh isolated MVs were double-stained with the fluorescein isothiocyanate (FITC)-conjugated isolectine B4 from *Bandeiraea simplicifolia* (Sigma Aldrich) and allophycocyanin (APC)-conjugated Annexin V (BioLegend, San Diego, CA, USA). A primary threshold of 6000 events was set at SSC-A parameter to avoid exclusion of the smallest events. A volume of 100 μl was recorded in each sample with a slow flow rate of 14 μL/min. IB4 and Annexin V single staining were included to allow the correct position of the MV gates. Non-specific staining was assessed using matched isotype controls.

CHME-5 cells were tested for the expression of myeloid markers (Supplementary Table 1). Briefly, cells were incubated in presence of the specific monoclonal antibodies in PBS containing 5% FBS for 30 min at 4 °C. Cells were then washed twice and resuspended with PBS before analysis on flow cytometer. Negative controls were performed by staining cells with isotype-matched control antibodies.

To analyse surface marker expression on MV subpopulations isolated from CHME-5 cells, MVs from different preparations were stained with the following monoclonal conjugated antibodies, PE/Cy5-conjugated CD11c, AF647-conjugated CD15, FITC-conjugated CD40, FITC-conjugated CD44, PE-conjugated CD63, PE-conjugated CD73, PE/Cy7-conjugated CD90, APC-conjugated CD146, APC-conjugated CD184, AF647-conjugated CCR6, PE-conjugated CD274 and PE-conjugated CX3CR1 (BioLegend). Samples were analysed at rates below 10,000 events/seconds and stopped when 1,000 events within the MV gate were collected. Expression levels of surface markers were quantified as the median fluorescence intensity (MFI) ratio calculated by dividing the MFI value of the specific antibody by the MFI value of the matched isotype control. Expression profiles of the twelve surface markers were used to perform a hierarchical cluster analysis. Data was mean-centered and log2 transformed before hierarchical clustering was obtained applying different metrics (Euclidean distance and Manhattan distance) and hierarchical clustering algorithms (UPGMA, WPGMA and WARD).

### Transmission electron microscopy (TEM)

Transmission electron microscopy (TEM) analysis were performed with a HT7700 Hitachi microscope (Tokyo, Japan). MV samples were prepared for TEM by negative staining method. A drop (10 μl) of MV suspension was placed onto glow discharged 200-mesh formvar/carbon copper grids (Electron Microscopy Sciences, Hatfield Township, PA). After 1 min, a drop (10 μl) of 1% phosphotungstic acid solution was added and left to act for 1 min to stain the sample. Then, the excess liquid was removed on the edge of the grid using filter paper. The resulting samples were dried in air at room temperature for 10 min and finally examined by electron microscopy. TEM images were analysed using Image J software (National Institutes of Health, USA) to measure MV size. At least 100 MVs from three independently prepared samples were quantify and used for size distribution analysis. All the images were prepared by setting the scale using scale bar, single MVs not touching/crossing one another were selected for analysis and their diameter measured.

### Atomic force microscopy (AFM)

The atomic force microscopy (AFM) measurements were performed by exploiting a Multimode 8 SPM coupled with a Nanoscope-V controller (Bruker-AXS, Santa Barbara, CA, USA). In order to minimize probe-sample interactions during scanning, the topographic images were obtained by tapping-mode AFM where a continuously oscillating probe is used to scan the sample. Briefly, mica coverslips were prepared by first mounting the entire mica stack onto a glass slide with two-sided tape. Scotch tape was applied to the top of the mica and then removed, which pulled off an entire layer of mica, leaving behind a freshly cleaved layer of mica on the glass slide. Therefore, 10 µL drops of MV suspension was deposited onto surface of clean layer of mica. Sample was air-dried at room temperature before of AFM measurements in a scan size of 5 microns square for each acquisition. The surface of a clean mica was used as background reference (data not shown). Images were collected from three different samples and were processed by Gwyddion 2.45 software. For each image, single MVs were analysed to obtain MV diameter. Clustered MVs and MVs with imaging artifacts were excluded from the analysis. MVs adjacent/touching to each other were included in the analysis only if separation between MVs can be clearly established. At least 50 MVs were measured to obtain MV size distribution.

### RNA isolation and detection

MV pellet samples were resuspendend in TRIzol reagent (Invitrogen) and RNA isolation was performed using Norgen's Single Cell RNA Purification Kit (Norgen Biotek) following the manufacturer’s recommendations. Isolated RNA was resuspended in RNase free water and RNA yield and quality was determined by using Qubit RNA HS Assay Kit on Qubit 2.0 Fluorometer (Thermo Scientific) according to the manufacturer’s protocol. RNA integrity and quality were analysed using the Agilent 2100 Bioanalyzer (Agilent Technologies, Santa Clara, CA) and the RNA 6000 Pico Kit.

### Protein quantification and Western blot analysis

Isolated MVs and exosomes were resuspended in lysis buffer (50 mM Tris, pH 7.5, 150 mM NaCl, 1% Triton X-100) in the presence of 0.1% SDS, supplemented with a protease inhibitor cocktail (Sigma-Aldrich). Protein concentration of the samples was measured using Micro-BCA (Thermo Scientific) and analysed by a Varian Cary 50 UV–Vis Spectrophotometer (Agilent Technologies). For each EV preparation, the corresponding cells and P1 pellet were recovered and solubilized in lysis buffer in presence of 0.5% SDS and protease inhibitor cocktail (Sigma-Aldrich). Cell and P1 lysates were incubated on ice for 30 min and then centrifuged at 10,000×*g* for 30 min at 4 °C. Protein concentrations were measured using the Protein Assay Dye Reagent Concentrate (Bio-Rad Laboratories) and analysed by a Varian Cary 50 UV–Vis Spectrophotometer (Agilent Technologies). Western blot analysis was performed according to standard procedure. Briefly, equal volumes or volumes containing equal amount of protein lysates from EV preparation (MVs and exosomes), P1 pellet and cells were diluted with Laemmli buffer (Bio-Rad Laboratories) with β-mercaptoethanol, heated to 95 °C for 5 min and separated on 4–15% Mini-PROTEAN TGX Stain-Free Precast Gels (Bio-Rad Laboratories). Proteins were transferred onto a Polyvinylidene Difluoride (PVDF) membrane with Trans-Blot Turbo Mini PVDF Transfer (Bio-Rad Laboratories). Membranes were blocked for 1 h with 5% Blotting Grade Blocker Non-Fat Dry Milk (Bio-Rad Laboratories) in PBS-Tween 0.1% and then probed with primary antibodies overnight at 4 °C (Supplementary Table 2). For chemiluminescence detection of proteins, HRP-linked anti-rabbit IgG and anti-mouse IgG (Cell Signaling Technology) secondary antibodies, and Clarity Max Western ECL Substrate were used. Signal were visualized using a ChemiDoc MP Imaging System (Bio-Rad Laboratories).

### Statistical analyses

Statistical analysis was carried out using one-way analysis of variance (ANOVA) or Student’s t-test for unpaired samples as appropriate. When indicate, Bonferroni post hoc tests were also performed. All analyses were performed using GraphPad Prism (version 5; GraphPad) software. Value of P < 0.05 was considered to be statistically significant.

## Results

### The choice of rotor type employed during the first centrifugation step of the isolation protocol affects the MV recovery

**Figure 1:**
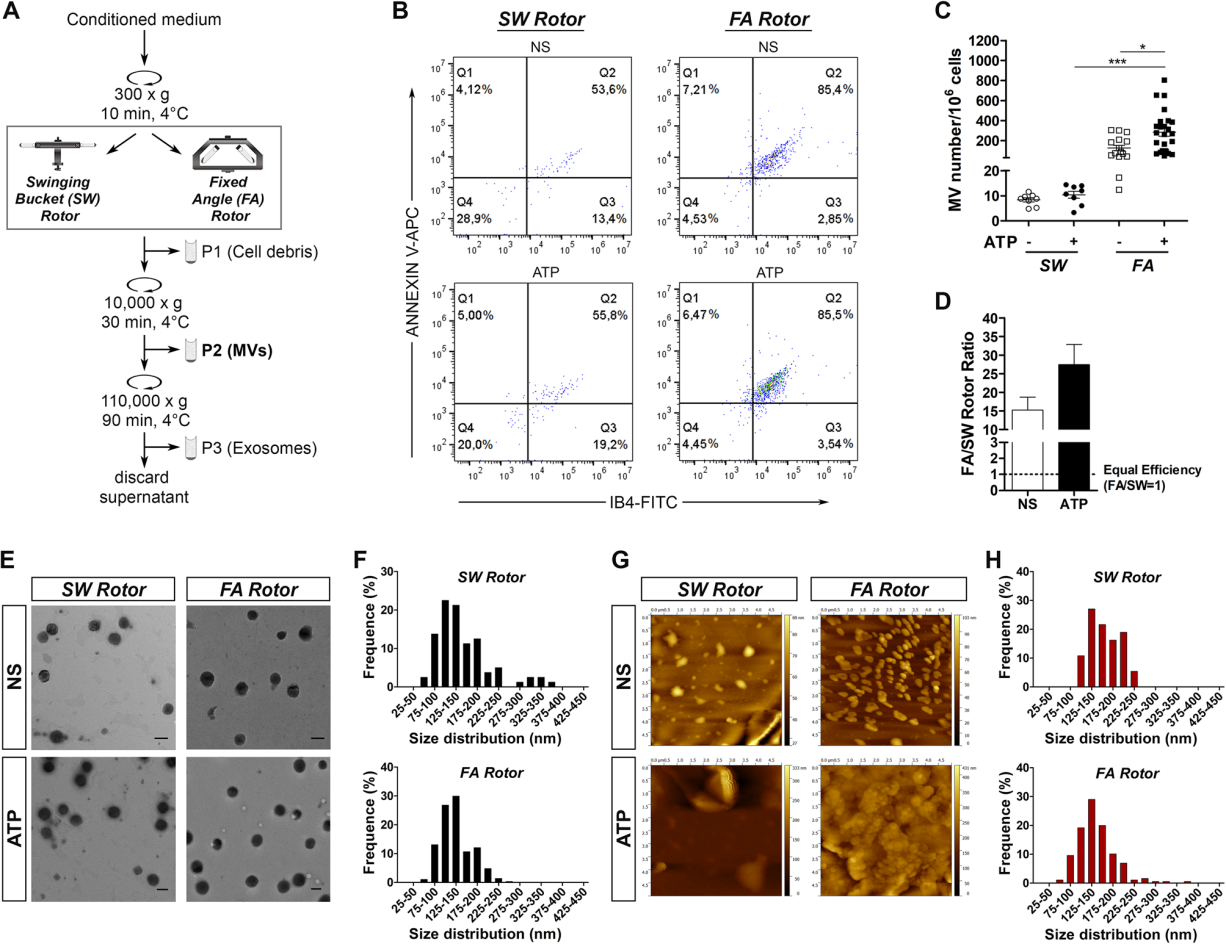
The rotor type employed during the first centrifugation step of the isolation protocol affects the MV recovery. (**A**) Flow chart of the differential centrifugation protocol. (**B**) Flow cytometry analysis (FlowJo, version 10) so of IB4/Annexin V double-stained MVs isolated from untreated (NS) and ATP-stimulated CHME-5 microglia cells adopting SW or FA rotor during the first centrifugation step. (**C**) Quantification of IB4/Annexin V + MVs isolated using the SW or the FA rotor in NS and ATP conditions as measured by flow cytometry. Data are presented as mean ± SEM, *p < 0.05, ***p < 0.001, ANOVA with Bonferroni post hoc test (GraphPad Prism, version 5). (**D**) FA to SW rotor ratio from MV quantification as shown in (**C**) calculated as (FA-MV number/10^6^ cells)/(SW-MV number/10^6^ cells). Data are reported as mean ± SEM (GraphPad Prism, version 5). (**E**, **G**) Transmission electron microscopy (TEM) and atomic force microscopy (AFM) of isolated MVs for SW and FA protocol, in NS and ATP conditions. Scale bars 200 nm. (**F**,**H**) Size distribution of isolated MVs in ATP condition determined from TEM (**F**, SW mean size of 155.4 ± 6.6 nm, n = 100; FA mean size of 133.8 ± 2.1 nm, n = 250) and AFM (**H**, SW mean size of 173 ± 11.5 nm, n = 50; FA mean size of 154.3 ± 2.8 nm, n = 300) images using Image J software.

Differential centrifugation protocols are commonly employed for the isolation and purification of the fraction of lEVs containing MVs. The typical protocol usually consists of a first centrifugation step at low-speed spin (e.g. 300×*g* for 10 min) to eliminate floating cells and cellular debris followed by a step of centrifugation at higher speed spin (e.g. 10,000×*g* for 30 min) to pellet MVs (Fig. 1A). The first (low-speed) centrifugation step is often regarded as a preliminary step that does not affect MV yield and/or quality. In this study, we evaluated how the first round of centrifugation has a critical effect on MV recovery. In particular, we evaluated the impact of applying the swinging-bucket (SW) or fixed-angle (FA) rotors during the first centrifugation step of MV isolation. MVs were isolated from cell-conditioned medium of the human CHME-5 embryonic microglia cells^29–31^ not stimulated (NS) or stimulated with ATP, a well-known molecule promoting microglia activation and MV release^32^, using the standard protocol described in Fig. 1A.

The lEVs recovered using the different rotor types were characterized and measured as bona fide MVs by flow cytometry after double-staining with isolectin IB4 (as marker of myeloid plasma membranes) and Annexin V (as marker of MV membranes) (Fig. 1B and Supplementary Fig. 1). The use of SW rotor in the first centrifugation step resulted in an embarrassing low recovery of MVs from the conditioned medium of microglia stimulated or not with ATP (Fig. 1B,C). On the other hand, the use of FA rotor significantly improved MV isolation efficiency and a significantly higher release of MVs were actually be observed in ATP stimulated microglia compared with NS cells (Fig. 1B,C). The increase of MV recovery observed employing FA rotor (calculated as the ratio of the MVs isolated using FA rotor to SW rotor) was of approximately 15–30 fold compared to SW rotor and occurred in both NS and ATP conditions (Fig. 1D; NS: 15.2 ± 3.5; ATP: 27.5 ± 5.4). Similar results were observed when the isolation protocol was employed to isolate MVs from the human THP-1 monocyte cells, thus showing that the use of different rotor types affects MV recovery independently from the cell type of origin (Supplementary Fig. 2). On the other hand, the use of SW rotor or FA rotor during the first round of isolation step did not affect the downstream isolation of small vesicles as no differences were observed in protein yields and exosomal markers between the exosome preparations obtained using the two different rotor types (Supplementary Fig. 3).

Transmission electron microscopy (TEM) and atomic force microscopy (AFM) were performed to validate MV preparations and characterize the morphology of MVs isolated using the two different rotor types. TEM and AFM analysis revealed no differences in morphology (Fig. 1E,G) and size (Fig. 1F,H) between MVs isolated using SW or FA rotor (Fig. 1F, TEM: 155.4 ± 6.6 nm for SW and 133.8 ± 2.1 nm for FA; Fig. 1H, AFM: 173 ± 11.5 nm for SW and 154.3 ± 2.8 nm for FA), thus suggesting that the use of the different rotors applied during the first step of the isolation protocol affects only the yield of isolated MVs but not their quality and morphology.

All the different fractions obtained during the differential centrifugation protocol employing the SW or the FA rotor (namely, conditioned medium (CM), P1 and P2; Fig. 1A) were analysed by flow cytometry and TEM to evaluate their content in MVs (Supplementary Fig. 4). Of note, the use of SW rotor during the first step of the isolation protocol causes the loss of a large number of vesicles in the discarded P1 pellet, where MVs appear entangled in a web of cellular debris. By contrast, the use of FA rotor allowed the efficient isolation of MVs in P2 pellet associated with the remarkable reduction of MVs in P1 fraction.

### An additional share of MVs is isolated employing a washing procedure to the first centrifugation step of the isolation protocol

**Figure 2:**
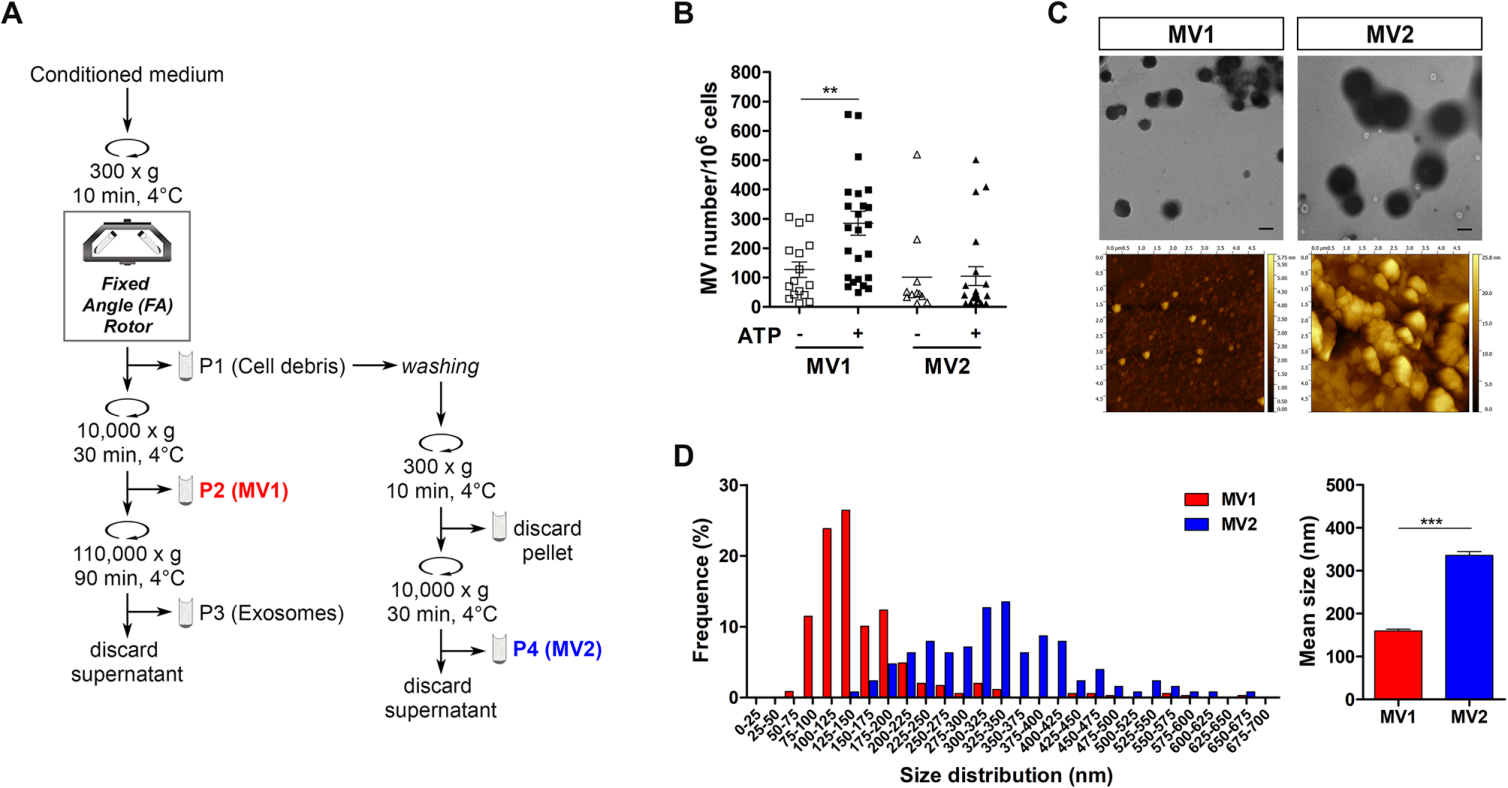
An additional washing step of the first discarded pellet enables isolation of a supplemental share of MVs from microglia cells. (**A**) Flow chart of an optimized differential centrifugation protocol including the additional washing step at the P1 pellet followed by a second MV isolation round. (**B**) Quantification of IB4/Annexin V + MVs recovered in MV1 and MV2 fractions as measured by flow cytometry. Data are reported as mean ± SEM, **p < 0.01, unpaired t-test (GraphPad Prism, version 5). (**C**) Transmission electron microscopy (TEM) and atomic force microscopy (AFM) of MV1 and MV2 collected fractions released from ATP-stimulated CHME-5 cells. Scale bars 200 nm. (**D**) Size distribution (left panel) and mean size (right panel) of MV1 and MV2 subpopulations determined from TEM image using Image J software (MV1 mean size of 159.4 ± 4.3 nm, n = 348; MV2 mean size of 335.4 ± 9.0 nm, n = 126). Data are reported as mean ± SEM, ***p < 0.001, unpaired t-test (GraphPad Prism, version 5).

To minimize the residual MV loss observed during the isolation procedure with FA rotor (Supplementary Fig. 4) a washing step of the usually discarded P1 pellet, followed by a second low-speed centrifugation, was added at the MV isolation protocol (Fig. 2A). The additional procedure allowed the recovery of the MVs entangled in the P1 fraction in a second MV pellet (P4-MV2; Fig. 2A) as assessed by flow cytometry after MV double-staining with IB4/Annexin V for both NS and ATP-stimulated conditions (Fig. 2B). Similar results were obtained when the additional procedure was employed to isolate MVs from the conditioned medium of untreated and ATP-stimulated THP-1 cells (Supplementary Fig. 5A,B), showing that an additional share of MVs can be isolated from P1 pellet regardless of the cell type.

The MVs isolated using the additional steps were validated by TEM and AFM analysis. The MV2 vesicles isolated from CHME-5 cells appeared to be structurally intact with a morphology similar to that of the MV1 vesicles group (Fig. 2C). However, according to TEM analysis, the MVs collected in the two pellets are selectively enriched by vesicles of different sizes (Fig. 2D). In particular, the MVs obtained in the first pellet (MV1) sized from 65 to 670 nm in diameter (mean size: 159.4 ± 4.3 nm) while the MV2 group contained larger vesicles with a size range from 135 to 650 nm (mean size: 335.4 ± 9.0 nm) (Fig. 2D). The isolation/identification of MVs with different sizes into the two distinct isolation fractions was specifically observed for CHME-5 cells while, the MVs isolated from THP-1 cells showed similar size distribution profiles with a diameter ranging from 140 to 990 nm (mean: 601.1 ± 17.8 nm) and from 125 to 900 nm (mean: 509.4 ± 13.2 nm) for the first and second fraction respectively (Supplementary Fig. 5C). Overall, these data suggest that (1) CHME-5 cells release MV subpopulations of discrete size, (2) the choice to apply or not an additional re-washing/centrifugation procedure to the P1 fraction can determine the selective loss of specific MV subpopulations.

### The microglia MV subpopulations selectively enriched into the two different isolation fractions show distinct molecular characteristics

**Figure 3:**
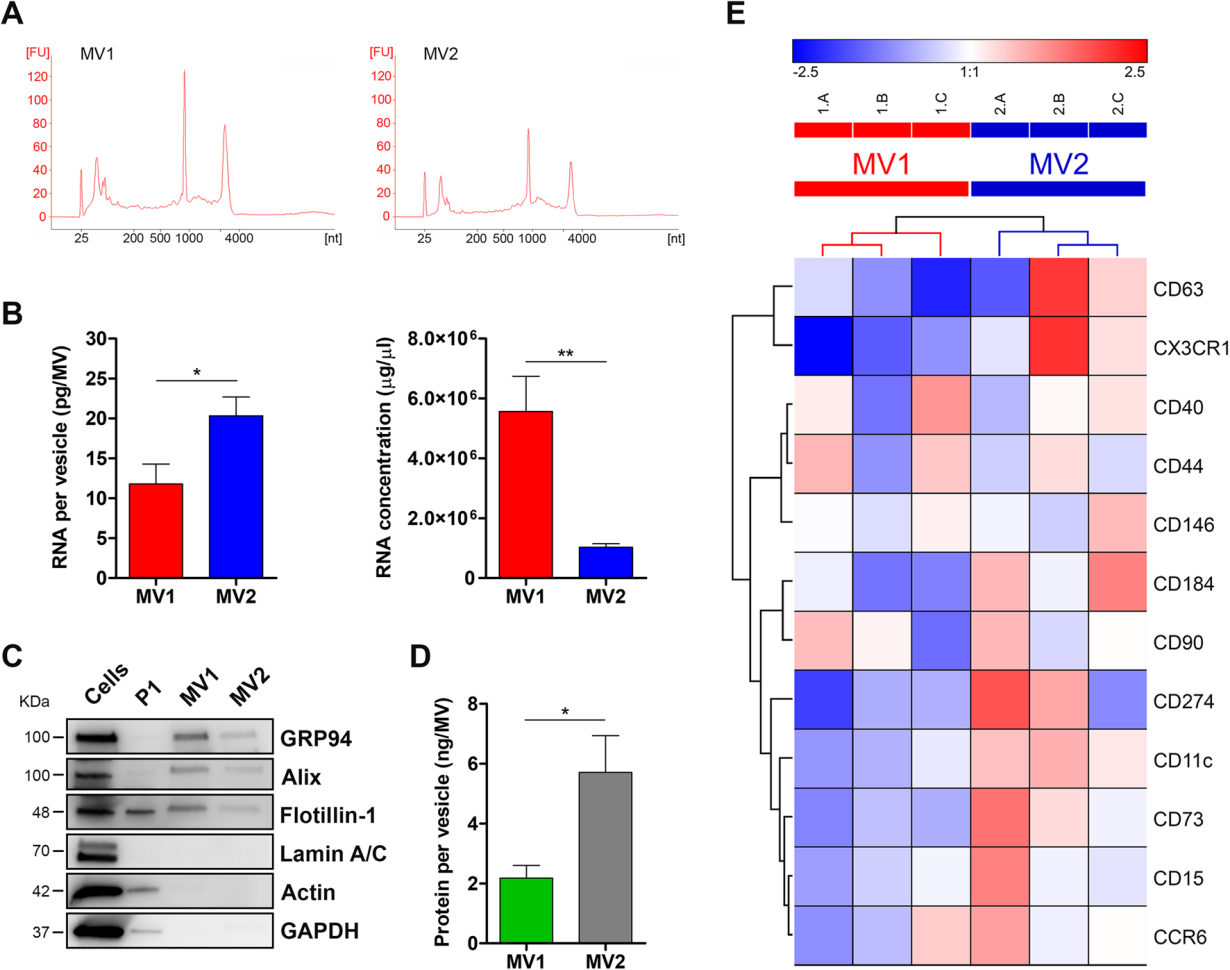
Molecular characterisation of the two MV fractions isolated from microglia cells. (**A**) Representative electropherograms showing profiles of RNA isolated from the two MV fractions analysed by Bioanalyzer (Agilent RNA 6000 Pico Assay; FU: fluorescence units). (**B**) RNA amount (pg/vesicle) and RNA concentration (µg/µl) quantified using Qubit 2.0 Fluorometer and Qubit RNA HS Assay Kit were graphed for each MV fraction. Data are presented as mean ± SEM, *p < 0.05, **p < 0.01, unpaired t-test (GraphPad Prism, version 5). (**C**) Western blot analysis of MV (GRP94, Alix and Flotillin-1), nuclear (Lamin A/C) and cytosolic (Actin and GAPDH) marker contents in MV1 and MV2 fractions and the corresponding CHME-5 cell lysate and P1 fraction. (**D**) Protein amount (ng/vesicle) quantified by micro-BCA assay was reported for each MV fraction. Data are presented as mean ± SEM, *p < 0.05, unpaired t-test (GraphPad Prism, version 5). (**E**) Hierarchical clustering analysis of the immunophenotypic flow cytometry data of MVs recovered in MV1 and MV2 fractions. The expression level of surface markers on MV membranes was measured as MFI ratio. Heatmap was rendered with Genesis 1.8.1 after mean centering and log2 normalization of the expression data. Each row represents a specific surface marker and each column represents individual MVs isolation. Boxes are coloured based on the level of expression of MV surface markers according to the colour scale at the top of the figure. Hierarchical clustering was obtained applying Euclidean distance and UPGMA (Unweighted Pair Group Method using Arithmetic mean) algorithm. Similar results were obtained with different metrics (e.g.: Manhattan distance) and hierarchical clustering algorithms (e.g.: WPGMA; WARD). The dendrogram of the MVs depicts a balanced tree in which the two MVs subpopulation are clearly separated and grouped into two distinct clusters.

To explore the differences between the two MV subpopulations identified in CHME-5 cells, the MVs of distinct sizes selectively enriched into the two fractions isolated from ATP-stimulated cells were analysed for their content in RNA cargo and proteins. Total RNA and proteins obtained from both fractions was mainly related/associated to MVs as shown by the direct correlation observed between the amounts of the purified RNA and proteins and the corresponding number of IB4/Annexin V + MVs isolated in MV1 and MV2 fractions (Supplementary Fig. 6; RNA, MV1: R = 0.8641, *p* < 0.05 and MV2: R = 0.9814, *p* < 0.0001; proteins, MV1: R = 0.9175, *p* < 0.01 and MV2: R = 0.7903, *p* < 0.0001). The RNA cargo of the two MV subpopulations was analysed using Agilent Bioanalyzer in order to compare their RNA profiles, since it is known that distinct MVs can contain and transport different RNA species (i.e. mRNA, ribosomal RNA, long and small non-coding RNA^33, 34^ (Fig. 3A). The MV subpopulations released by CHME-5 cells showed very similar RNA profiles with the three dominant peaks corresponding to rRNAs (28S and 18S) and small RNAs (50–200 nt). However, the two different-sized MV subpopulations were found to contain/transport significantly different amounts of RNA. The MV2 subpopulation consisting of large vesicles contained more RNA per vesicles compared to the MV1 subpopulation of small vesicles. On the other hand, RNA concentration was significantly higher in the small vesicles than in the large vesicles (Fig. 3B). The two MV subpopulations released by CHME-5 cells were then characterized by Western blot for the presence of established microvesicles markers. MV markers, such as GRP94, Alix and Flotillin-1 were expressed/enriched in both MV subpopulations. On the other hand, Lamin A/C (nuclear marker), Actin and GADPH (cytosolic markers) were absent in MV fractions and were barley present in discarded P1 fraction confirming that the two isolated MV subpopulations contained little or no contamination of cellular components (Fig. 3C and Supplementary Fig. 7). These results indicated that both MV subpopulations express similar protein markers. However, as observed for RNA, the protein content per vesicle was significantly higher in the large vesicles (MV2 subpopulation) compared to the small vesicles (MV1 subpopulation) (Fig. 3D).

To further delineate the different nature of the two MV subpopulations derived from CHME-5 cells the expression profiles of their surface markers were investigated using a flow cytometry-based approach. A panel of twelve myeloid surface markers specifically expressed by CHME-5 cells (Supplementary Fig. 8 and Supplementary Table 1) was analysed for their expression in MV subpopulations, as it is known that MVs express on their membranes the same surface markers of their parental cells^35^. The expression level of each specific surface marker on MV membranes was measured as the mean fluorescence intensity (MFI) ratio, which was obtained dividing the MFI determined for a specific antibody by the MFI of the appropriate isotype control (Supplementary Fig. 9). MV1 and MV2 microvesicles expressed different levels of surface markers with a majority of markers being more expressed in MV2 subpopulation (Fig. 3E). Moreover, hierarchical clustering analysis based on expression of the twelve surface markers clearly clustered the two MV subpopulations in two distinct groups. Overall, these results suggest that CHME-5 cells release two distinct MV subpopulations differing in terms of dimensions, RNA content and surface marker expression.

## Discussion

The high heterogeneity of EVs, in terms of size and composition, as well as their nanoscale dimensions, pose a number of limits and challenges to their effective isolation and purification^36–38^. To date, differential centrifugation remains the gold-standard technique for EVs (MVs and exosomes) isolation and it is routinely used in many laboratories to isolate EVs from both cell conditioned media and body fluids^11, 39^. However, the yield and purity of the EVs isolated by differential centrifugation are strongly affected by the different factors and parameters employed to the protocol (e.g.: rotor type, centrifugal force, centrifugation time and temperature) and EV preparations suffer from vesicle aggregation and protein contamination that influence and compromise the accuracy of the downstream analyses^20, 24, 40, 41^. The limits and ability of the differential centrifugation protocols to actually isolate and purify EVs have been largely investigated for exosomes but remain poorly explored for MVs^28, 42–44^. In this study, we examined in detail the capacity of a classical protocol of differential centrifugation to specifically isolate MVs from conditioned medium of human cell lines. In particular, we demonstrated that the choice of rotor type to be used during the first step of differential centrifugation protocol (FA *vs* SW) make the difference between a good or a bad MV preparation. Several studies had already investigated the critical role played by the different rotor types during the final centrifugation steps of the isolation protocol on EVs (exosomes) purification^20, 25^. On the other hand, the first low-speed centrifugation step of the differential isolation protocol (designed to remove dead cells and cell debris) was usually regarded as uninfluential on EV isolation and, to the best of our knowledge, the impact of using different rotor types during this step of the isolation protocol on MVs and/or exosomes recovery has never been investigated. Here, we revealed that this first centrifugation step is the key point of the MV isolation protocol. It is long known that during the first centrifugation step of a differential centrifugation protocol, small particles (as MVs) can remain entrapped in the discarding pellet causing the loss of the small particles form the supernatant fraction and the contamination of the related pellet^45^. We observed that the application of SW rotor during the low-speed centrifugation strongly enhanced this drawback leading to the loss of a large number of vesicles in the discarded pellet and poor MV preparations. On the other hand, the use of FA rotors in the first centrifugation step can to some degree overcome this drawback allowing to significantly increase the amount of isolated vesicles. Interestingly, the downstream isolation of the exosomes is not affected by this drawback likely because, exosomes, due to their smaller size, can more easily escape/slip through the web of cellular debris.

The different amount of MVs entrapped in the first pellet of the isolation protocol is most likely due to the different geometry of the rotors employed during the first centrifugation step. In fact, as a result of the rotor design, particles (cells, debris and/or MVs) sediment directly to the tube bottom in SW rotors while, in FA rotors, particles sediment over the extended area along the outer tube wall and then slide down to the tube bottom. As a consequence, the area of sedimentation is generally larger in FA rotors than in SW rotors which might result in less interactions between cell debris and MVs that lead to a reduction of MV entrapment/loss in first low-speed pellet. Moreover, in the FA rotors, the sliding of particles along the outer wall of the tube generates a convective flow from the top to the bottom of the tube that might exert a disentangling action on the MVs trapped in the cellular debris limiting their loss.

To prevent the loss of MVs in the low-speed pellet and increase MV recovery we added a washing step of the first pellet to the MV isolation protocol. To this aim, we adapted a standard washing procedure (consisting of pellet resuspension and re-centrifugation) employed at the end of the centrifugation steps of the classical differential centrifugation protocols to reduce pellet contamination^45^. The application of this simple washing procedure to the low-speed pellet followed by a second MV isolation allowed the collection of an additional share of MVs and increased the overall MV recovery yield independently of the cell type investigated (CHME-5 and THP-1). Unexpectedly, the use of the isolation protocol comprehensive of the washing procedure of first pellet led to collect/distribute microglia-CHME-5 (but not monocyte-THP-1) derived MVs according to size throughout the two isolation fractions. In particular, small-sized MVs were preferentially collected in the first MV pellet of the isolation protocol, while large-sized MVs were principally retrieved in the MV pellet obtained after the washing procedure. This peculiar behaviour is likely due to the co-occurrence of two different factors: (1) the diverse entrapment of the MVs of different sizes into the first discarded pellet and (2) the release of two distinct MV subpopulations of different sizes by microglia cells. In fact, it can be expected that, during the isolation procedure, large-size MVs are more likely to become entrapped in the first discarded pellet than small-size MVs. Accordingly, when the MVs to be isolated consist of two subpopulations of different sizes, the size-dependent entrapment of MVs leads to collect the major part of the small-MV subpopulation in the first MV pellet/fraction and the major part of the large-MV subpopulation in the MV pellet/fraction obtained after the washing procedure.

MVs are assumed to be a heterogeneous population of EVs, however, to date, few studies have specifically investigated the heterogeneity existing within this secreted EV population^28, 44^. The two microglia MV subpopulations selectively isolated/enriched into the two different MV fractions differed not only in sizes but also in the amounts of the transported/contained RNA and proteins and in the expression profiles of their surface markers. These observations confirm that CHME-5 microglia cells release two different MV subpopulations with distinct structural and biological features and provide further evidence of the heterogeneity of MVs.

Typically, validation and optimization of EV isolation protocols (MVs included) analysed the final step(s) of the protocols and were focused on reducing/eliminating the cross-contamination among EV populations/subpopulations, a common and inevitable drawback of the isolation procedures^46, 47^. On the other hand, the critical (r)evaluations of the EV isolation protocols have completely disregarded the eventuality that specific/distinct subpopulations of EVs/MVs could be lost during the isolation procedures. We showed that the unintentional loss of specific MV subpopulations represent an actual and underestimate problem of MV isolation methods. In this respect, our results highlight the fundamental necessity to (1) validate the effective capacity of the isolation procedures to capture/isolate the heterogeneity within the MV population and (2) optimize the MV isolation protocols in order to avoid the selective loss of specific MV subpopulations that can compromise downstream analysis, data acquisition and interpretations.

In conclusion, in this study we analysed the limits and the actual capacity of the differential centrifugation protocols for the isolation of MVs. We demonstrated that the usually mistreated first low-speed centrifugation step of the differential centrifugation protocol plays a crucial role for MV isolation and that the specific parameters and conditions applied to this step (i.e. rotor types and first pellet re-washing) strongly affect the yield and the quality of MV preparations. Despite the lack of a standardized protocol of differential centrifugation for the isolation of MVs, this method remains the most used technique for MV isolation. We proposed an optimized differential centrifugation isolation protocol and defined critical steps, parameters and procedures that should be accurately evaluated and routinely employed into the MV isolation procedures to avoid poor quality preparations that can undermine the analysis and characterization of MVs and the understanding of their biological and physiological roles.

## Supplementary Information

Supplementary Information 1.

## References

[1] Camussi G, Deregibus MC, Bruno S, Cantaluppi V, Biancone L (2010). Exosomes/microvesicles as a mechanism of cell-to-cell communication. Kidney Int..

[2] Théry C, Ostrowski M, Segura E (2009). Membrane vesicles as conveyors of immune responses. Nat. Rev. Immunol..

[3] Yáñez-Mó M (2015). Biological properties of extracellular vesicles and their physiological functions. J. Extracell. Vesicles.

[4] Nigro, A. *et al.* Myeloid extracellular vesicles: Messengers from the demented brain. *Front. Immunol.* **7** (2016). 10.3389/fimmu.2016.00017 PMC4731486 26858720

[5] Meldolesi J (2018). Exosomes and ectosomes in intercellular communication. Curr. Biol..

[6] Cufaro MC (2019). Extracellular vesicles and their potential use in monitoring cancer progression and therapy: The contribution of proteomics. J. Oncol..

[7] van der Pol E, Böing AN, Harrison P, Sturk A, Nieuwland R (2012). Classification, functions, and clinical relevance of extracellular vesicles. Pharmacol. Rev..

[8] Raposo G, Stoorvogel W (2013). Extracellular vesicles: Exosomes, microvesicles, and friends. J. Cell Biol..

[9] Colombo M, Raposo G, Théry C (2014). Biogenesis, secretion, and intercellular interactions of exosomes and other extracellular vesicles. Annu. Rev. Cell Dev. Biol..

[10] Cocucci E, Meldolesi J (2015). Ectosomes and exosomes: Shedding the confusion between extracellular vesicles. Trends Cell Biol..

[11] Théry C (2018). Minimal information for studies of extracellular vesicles 2018 (MISEV2018): a position statement of the International Society for Extracellular Vesicles and update of the MISEV2014 guidelines. J. Extracell. Vesicles.

[12] Zaborowski MP, Balaj L, Breakefield XO, Lai CP (2015). Extracellular vesicles: Composition, biological relevance, and methods of study. Bioscience.

[13] van Niel G, D’Angelo G, Raposo G (2018). Shedding light on the cell biology of extracellular vesicles. Nat. Rev. Mol. Cell Biol..

[14] Gardiner C (2016). Techniques used for the isolation and characterization of extracellular vesicles: Results of a worldwide survey. J. Extracell. Vesicles.

[15] Szatanek R, Baran J, Siedlar M, Baj-Krzyworzeka M (2015). Isolation of extracellular vesicles: Determining the correct approach (Review). Int. J. Mol. Med..

[16] Konoshenko MYu, Lekchnov EA, Vlassov AV, Laktionov PP (2018). Isolation of extracellular vesicles: General methodologies and latest trends. BioMed Res. Int..

[17] Coumans FAW (2017). Methodological guidelines to study extracellular vesicles. Circ. Res..

[18] Théry, C., Amigorena, S., Raposo, G. & Clayton, A. Isolation and characterization of exosomes from cell culture supernatants and biological fluids. *Curr. Protoc. Cell Biol.* **30**, 3.22.1–3.22.29 (2006). 10.1002/0471143030.cb0322s30 18228490

[19] Witwer KW (2013). Standardization of sample collection, isolation and analysis methods in extracellular vesicle research. J. Extracell. Vesicles.

[20] Cvjetkovic A, Lötvall J, Lässer C (2014). The influence of rotor type and centrifugation time on the yield and purity of extracellular vesicles. J. Extracell. Vesicles.

[21] Van Deun J (2014). The impact of disparate isolation methods for extracellular vesicles on downstream RNA profiling. J. Extracell. Vesicles.

[22] Taylor DD, Shah S (2015). Methods of isolating extracellular vesicles impact down-stream analyses of their cargoes. Methods.

[23] Kalra H (2013). Comparative proteomics evaluation of plasma exosome isolation techniques and assessment of the stability of exosomes in normal human blood plasma. Proteomics.

[24] Lobb RJ (2015). Optimized exosome isolation protocol for cell culture supernatant and human plasma. J. Extracell. Vesicles.

[25] Livshits MA (2015). Isolation of exosomes by differential centrifugation: Theoretical analysis of a commonly used protocol. Sci. Rep..

[26] Baranyai T (2015). Isolation of exosomes from blood plasma: Qualitative and quantitative comparison of ultracentrifugation and size exclusion chromatography methods. PLoS ONE.

[27] Jeppesen DK (2014). Comparative analysis of discrete exosome fractions obtained by differential centrifugation. J. Extracell. Vesicles.

[28] Kowal J (2016). Proteomic comparison defines novel markers to characterize heterogeneous populations of extracellular vesicle subtypes. Proc. Natl. Acad. Sci..

[29] Janabi N, Peudenier S, Héron B, Ng KH, Tardieu M (1995). Establishment of human microglial cell lines after transfection of primary cultures of embryonic microglial cells with the SV40 large T antigen. Neurosci. Lett..

[30] Colombo F (2018). Cytokines stimulate the release of microvesicles from myeloid cells independently from the P2X7 receptor/acid sphingomyelinase pathway. Front. Immunol..

[31] Vergara D (2019). Distinct protein expression networks are activated in microglia cells after stimulation with IFN-γ and IL-4. Cells.

[32] Bianco F (2005). Astrocyte-derived ATP induces vesicle shedding and IL-1β release from microglia. J. Immunol..

[33] Crescitelli R (2013). Distinct RNA profiles in subpopulations of extracellular vesicles: Apoptotic bodies, microvesicles and exosomes. J. Extracell. Vesicles.

[34] Lunavat TR (2015). Small RNA deep sequencing discriminates subsets of extracellular vesicles released by melanoma cells—evidence of unique microRNA cargos. RNA Biol..

[35] Lukacs-Kornek, V., Julich-Haertel, H., Urban, S. K. & Kornek, M. Multi-surface antigen staining of larger extracellular vesicles. in *Extracellular Vesicles* (eds. Kuo, W. P. & Jia, S.) vol. 1660 201–208 (Springer New York, 2017). 10.1007/978-1-4939-7253-1_16 28828658

[36] Willms E, Cabañas C, Mäger I, Wood MJA, Vader P (2018). Extracellular vesicle heterogeneity: subpopulations, isolation techniques, and diverse functions in cancer progression. Front. Immunol..

[37] Lässer C, Jang SC, Lötvall J (2018). Subpopulations of extracellular vesicles and their therapeutic potential. Mol. Aspects Med..

[38] Chiriacò M (2018). Lab-on-chip for exosomes and microvesicles detection and characterization. Sensors.

[39] Momen-Heravi, F. Isolation of Extracellular Vesicles by Ultracentrifugation. in *Extracellular Vesicles* (eds. Kuo, W. P. & Jia, S.) vol. 1660 25–32 (Springer New York, 2017). 10.1007/978-1-4939-7253-1_3 28828645

[40] Linares R, Tan S, Gounou C, Arraud N, Brisson AR (2015). High-speed centrifugation induces aggregation of extracellular vesicles. J. Extracell. Vesicles.

[41] Nordin JZ (2015). Ultrafiltration with size-exclusion liquid chromatography for high yield isolation of extracellular vesicles preserving intact biophysical and functional properties. Nanomed. Nanotechnol. Biol. Med..

[42] Willms E (2016). Cells release subpopulations of exosomes with distinct molecular and biological properties. Sci. Rep..

[43] Jeppesen DK (2019). Reassessment of exosome composition. Cell.

[44] Crescitelli R (2020). Subpopulations of extracellular vesicles from human metastatic melanoma tissue identified by quantitative proteomics after optimized isolation. J. Extracell. Vesicles.

[45] Ohlendieck, K. & Harding, S. E. Centrifugation and Ultracentrifugation. in *Wilson and Walker’s Principles and Techniques of Biochemistry and Molecular Biology* (2018).

[46] Tauro BJ (2012). Comparison of ultracentrifugation, density gradient separation, and immunoaffinity capture methods for isolating human colon cancer cell line LIM1863-derived exosomes. Methods.

[47] Ramirez MI (2018). Technical challenges of working with extracellular vesicles. Nanoscale.

