# Supplementary material for: Selective loss of microvesicles is a major issue of the differential centrifugation isolation protocols
Source: Sci Rep. 2021 Feb 11;11:3589. doi: 10.1038/s41598-021-83241-w (PMC7878808; doi:10.1038/s41598-021-83241-w)
Supplement: Supplementary file 1 — Supplementary Information 1. [file 41598_2021_83241_MOESM1_ESM.pdf]

Supplementary Material

Selective loss of microvesicles is a major issue of the differential centrifugation isolation protocols.

Annamaria Nigro[1], Annamaria Finardi[1], Marzia M. Ferraro[2], Daniela E. Manno[3], Angelo Quattrini[1], Roberto Furlan[1], Alessandro Romano[1*].

[1] Division of Neuroscience, Institute of Experimental Neurology, San Raffaele Scientific Institute, Milano, Italy.
[2] Department of Biological and Environmental Sciences and Technologies, University of Salento, Lecce, Italy.
[3] Department of Mathematics and Physics "E. De Giorgi", University of Salento, Lecce, Italy.

*Corresponding author: Alessandro Romano.
Address: Division of Neuroscience, Institute of Experimental Neurology, San Raffaele Scientific Institute, Via Olgettina, 60, 20132 Milan, Italy.
Phone: +39 0832 319302.
e-mail: romano.alessandro@hsr.it

Supplementary Figure 1

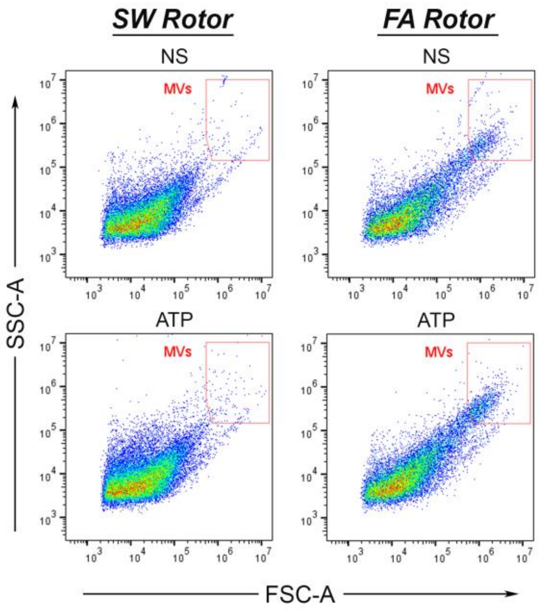

Figure S1. Flow cytometry gating strategy for CHME-5 derived MVs. Representative forward (FSC) versus side scatter (SSC) plots of MVs isolated from the conditioned medium of microglia (CHME-5) cells stimulated or not with ATP using the SW or FA rotor during the first centrifugation step of the isolation protocol.

Supplementary Figure 2

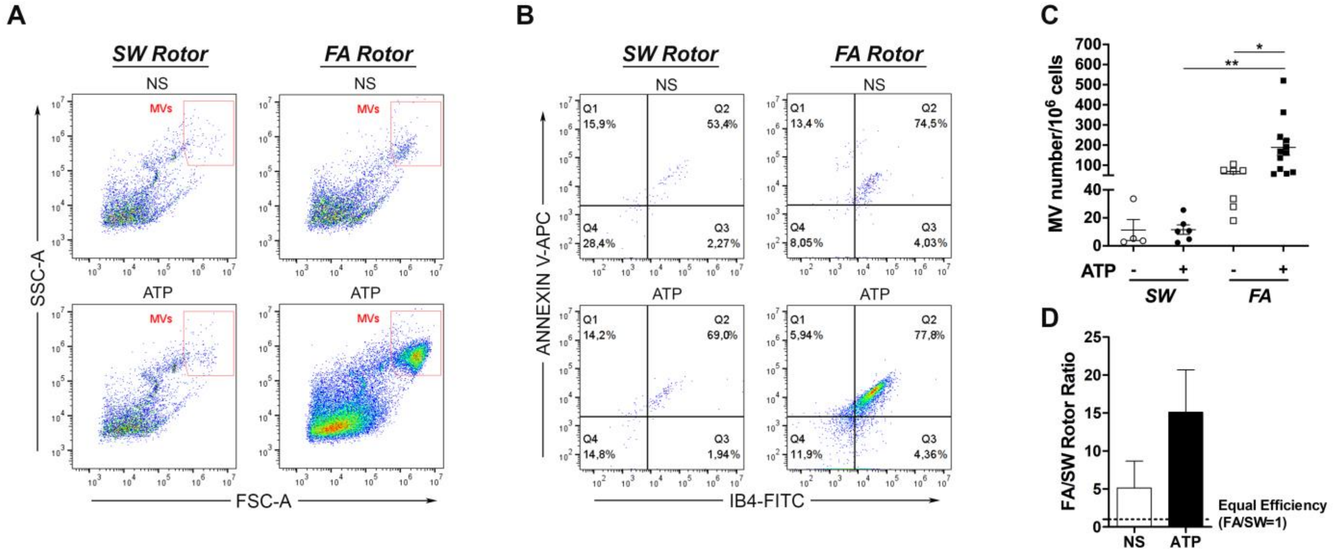

Figure S2. Flow cytometry analysis of MVs isolated from the human monocyte cell line THP-1. (**A**) MVs released from untreated (NS) or ATP-stimulated THP-1 cells are visualized on forward (FSC) versus side scatter (SSC) plots to gate MV population. (**B**) MVs are characterized for the double-staining with isolectin IB4 and Annexin V. Data show percentage of positive MVs. (**C**) Flow cytometry counts of IB4/Annexin V+ MVs isolated using SW or FA rotor, in untreated and ATP-stimulated conditions. Data are reported as mean ± SEM, *$p<0.05$, **$p<0.01$, ANOVA with Bonferroni post hoc test. (**D**) FA to SW rotor ratio from MV quantification shown in (C), calculated as (MV number FA/10^6 cells)/(MV number SW/10^6 cells). Data are presented as mean ± SEM.

Supplementary Figure 3

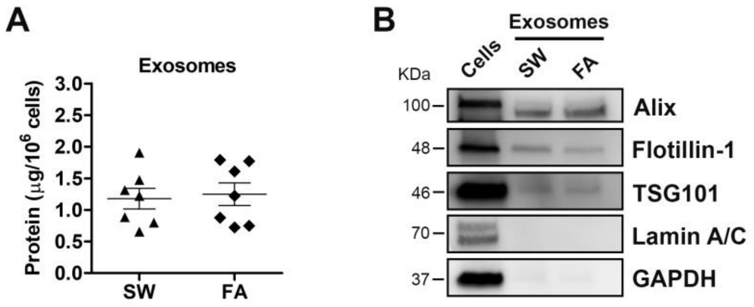

Figure S3. The rotor type employed during the first centrifugation step of the isolation protocol does not affect the exosome recovery. (**A**) Protein content analysis of the exosomes (P3 pellet) isolated from ATP-stimulated CHME-5 cells using SW or FA rotor during the first centrifugation step. Data are presented as microgram (µg) protein/10^6 cells with mean ± SEM. (**B**) Western blot analysis of the exosome (Alix, Flotillin-1 and TSG101), nuclear (Lamin A/C) and cytosolic (GAPDH) marker contents in exosome fractions isolated using the SW or the FA rotor and CHME-5 cell lysate. Equal protein amounts of the exosome factions were loaded on SDS-PAGE gels.

Supplementary Figure 4

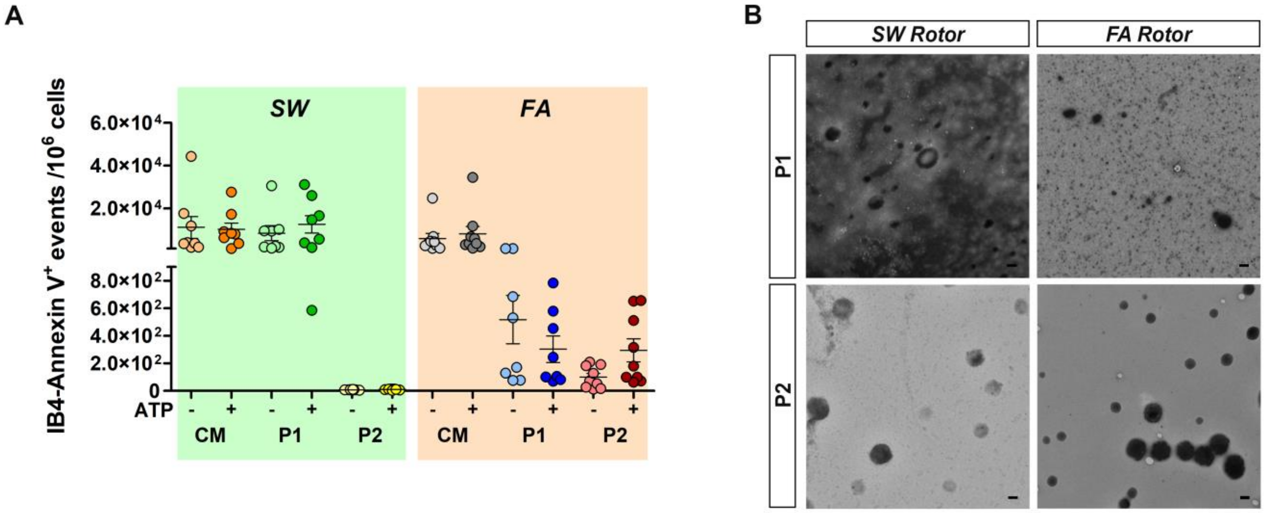

Figure S4. Analysis of the different fractions obtained from CHME-5 cells using the differential centrifugation protocol with SW or FA rotor in the first centrifugation step. (**A**) Flow cytometry counts of IB4/Annexin V+ events in cell conditioned media (CM), P1 and P2 pellets obtained using the SW or FA rotor, for untreated and ATP stimulated conditions. Data are reported as mean ± SEM (P2, SW-NS *vs* FA-NS, p=0,0056; P2, SW-ATP *vs* FA-ATP p= 0,0059; P2, FA-NS *vs* FA-ATP p= 0,0404, unpaired t-test). (**B**) Representative TEM images of P1 and P2 fractions isolated from supernatants of ATP stimulated CHME-5 cells, for both SW and FA rotor. Scale bars, 200 nm.

Supplementary Figure 5

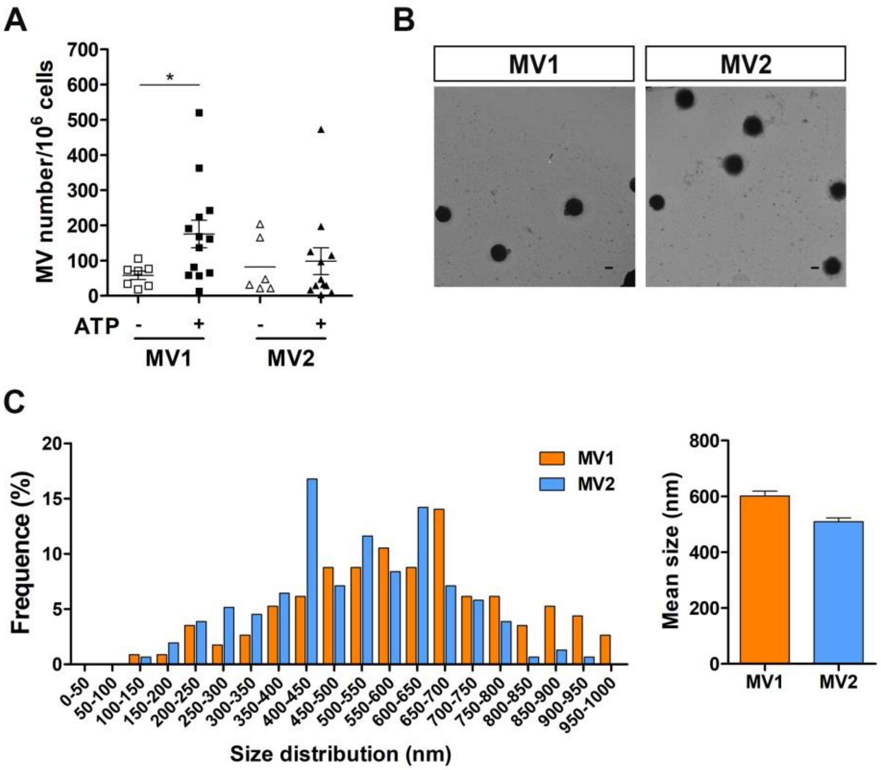

Figure S5. The additional washing step of the first discarded pellet allows isolation of two MV fractions from THP-1 monocyte cells with similar size distribution profiles. (**A**) Quantification of IB4/Annexin V+ MVs recovered in MV1 and MV2 fractions as measured by flow cytometry. Data are reported as mean ± SEM, *p<0.05, unpaired t-test. (**B**) Representative TEM images of MVs isolated in MV1 and MV2 fractions from conditioned media of ATP-treated cells. Scale bars, 200 nm. (**C**) Size distribution (left panel) and mean size values (right panel) of MV1 and MV2 subpopulations estimated from TEM analysis measurements (mean size of 601.1 ± 17.8 nm for MV1, n=114; mean size of 509.4 ± 13.2 nm for MV2, n=155). Data are reported as mean ± SEM.

Supplementary Figure 6

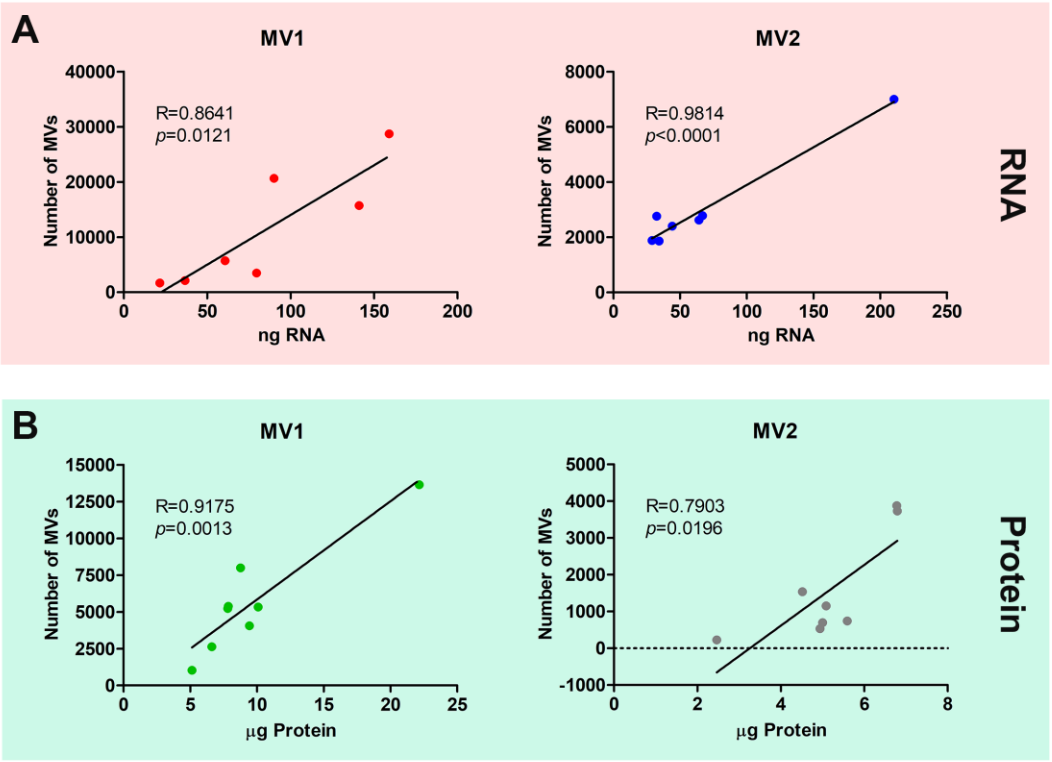

Figure S6. Correlation between the purified RNA/protein content and the number of isolated MVs for the MV1 and MV2 fractions of microglia cells. The graphs show the direct correlation between the amount of RNA (**A**) and protein (**B**) and the number of IB4/Annexin V+ MVs as measured by flow cytometry for both MV1 and MV2 fractions. Regression line and Pearson's correlation coefficient are shown.

Supplementary Figure 7

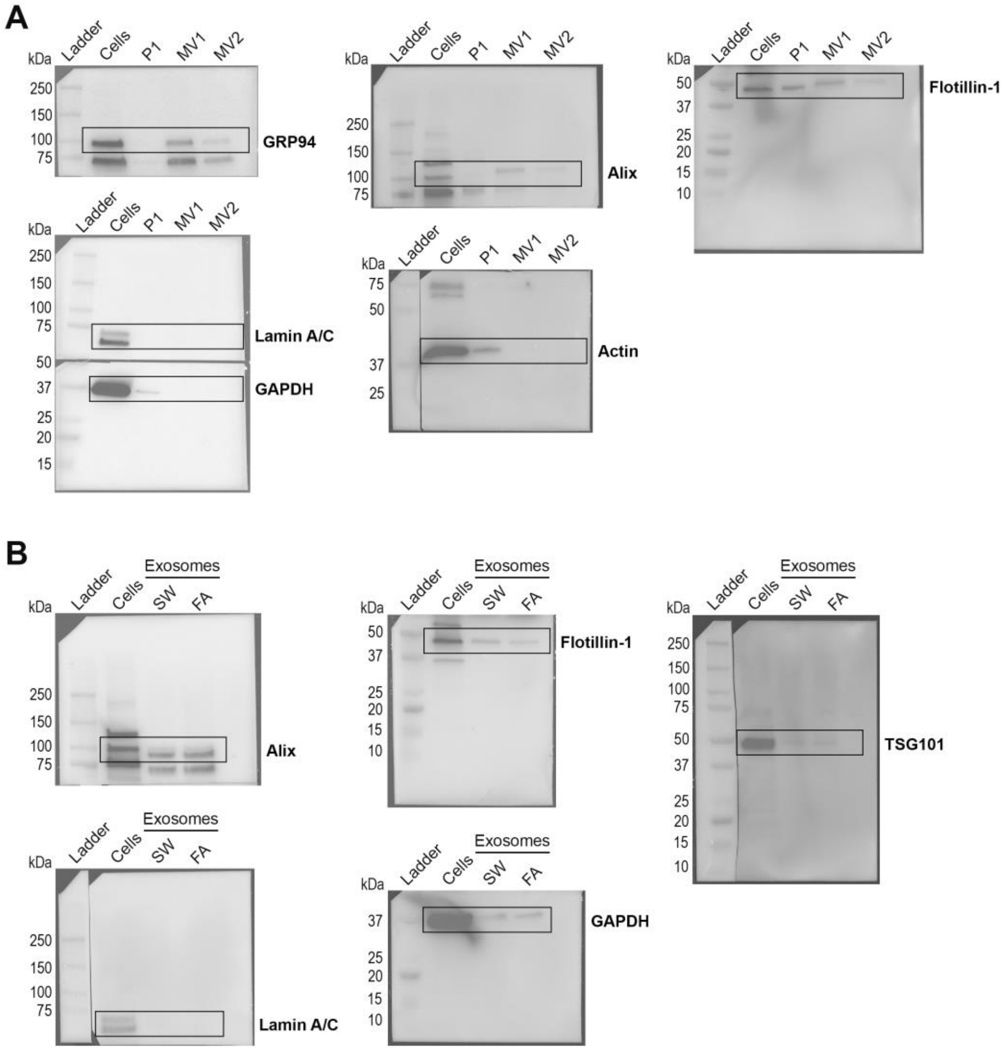

Figure S7. Original Western blots used for the main Figures. Original blots corresponding to Figure 3D (**A**) and Figure S3 (**B**). For each blot, a rectangle defines the area corresponding to that showed in the main figure. Signals were visualized using a ChemiDoc MP Imaging System (Bio-Rad Laboratories). Colorimetric and chemiluminescent images were merged to show ladder and protein detection in the same image.

Supplementary Figure 8

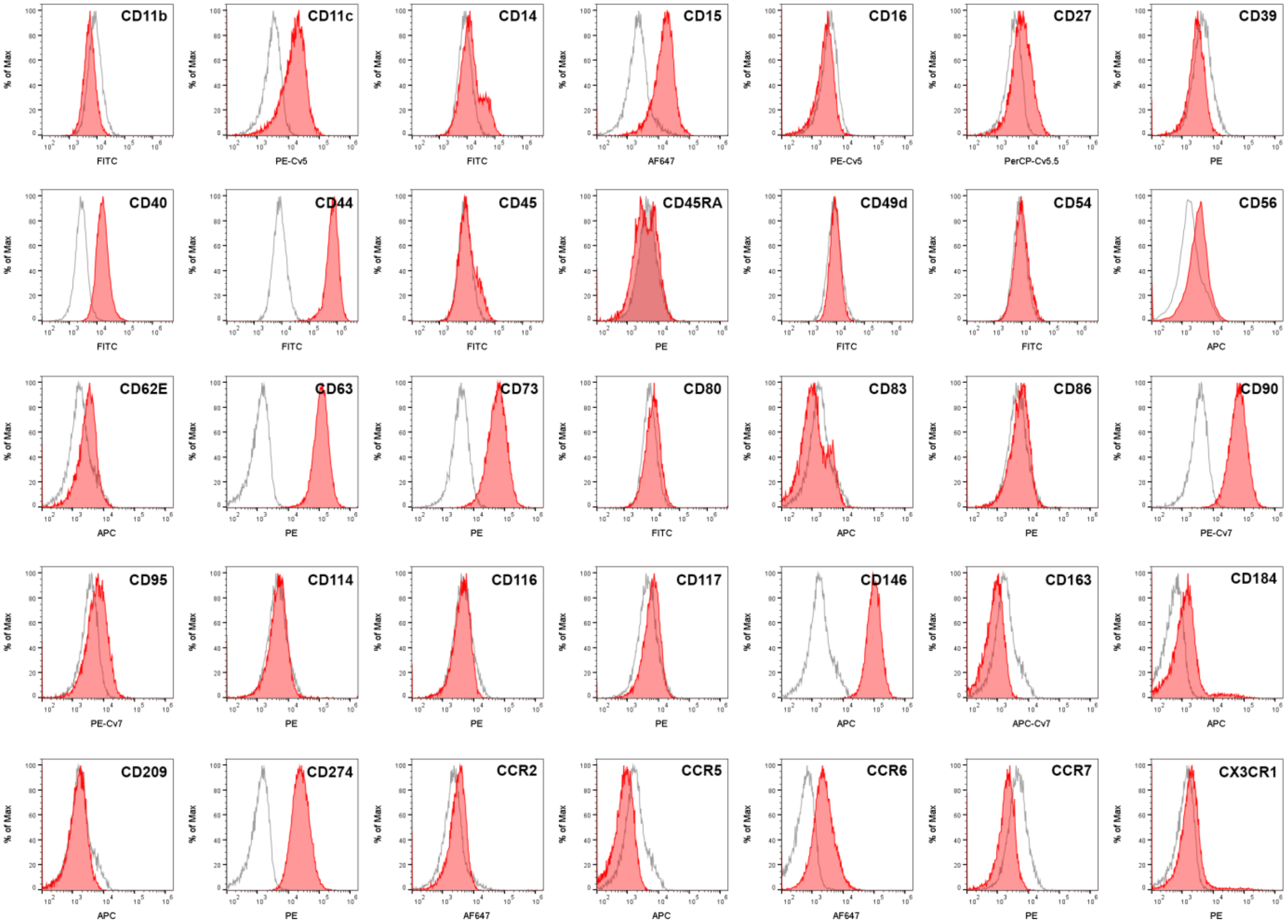

Figure S8. Flow cytometric identification of cell surface markers on ATP-stimulated CHME-5 microglia cells. Representative flow cytometry plots show the expression of myeloid cell surface markers. Filled histograms (in red) represent the cell surface antigens, the grey histograms represent the background staining obtained with the isotype-matched control antibody.

Supplementary Figure 9

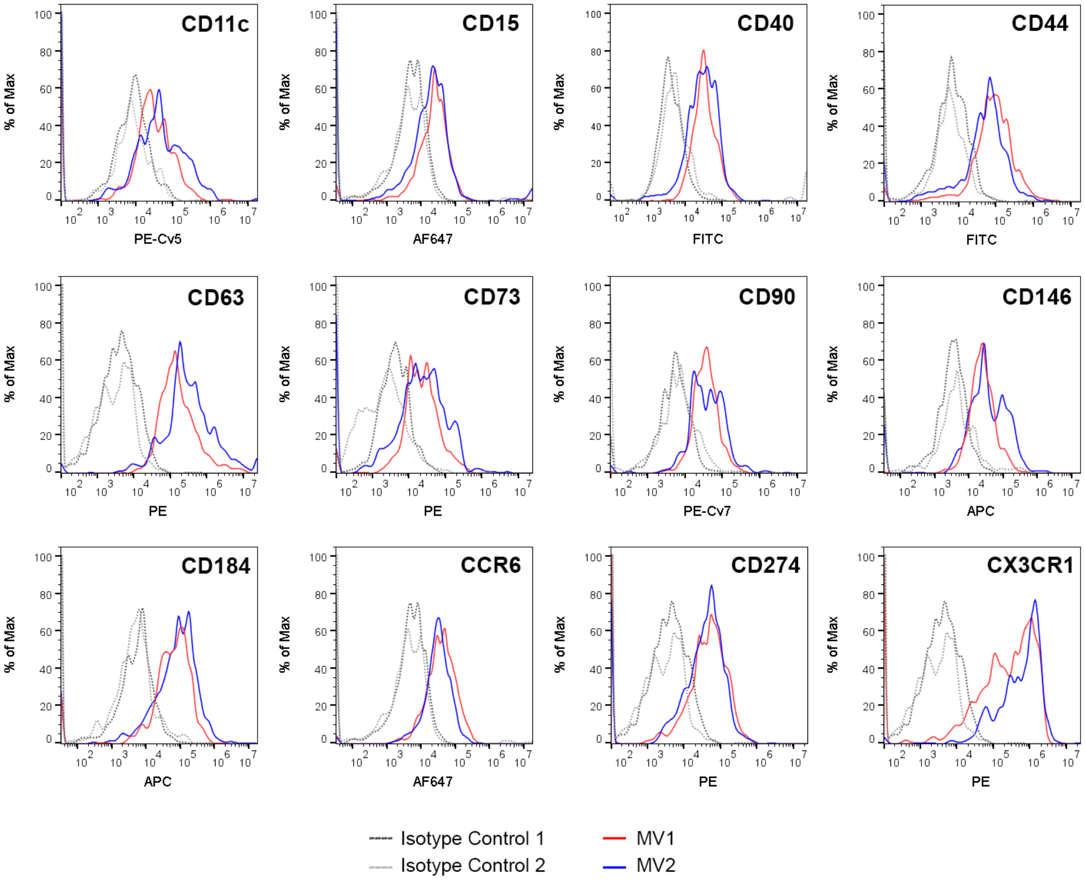

Figure S9. Flow cytometry analysis of cell surface marker expression in microglia-derived MV subpopulations. Representative flow cytometry plots show the expression levels of selected surface markers in MV1 (red line) and MV2 (blue line) subpopulations of MVs isolated from ATP-stimulated CHME-5 cells. Grey dotted lines represent the matched isotype controls. Graphs correspond to one representative of three independent experiments.

Supplementary Table 1. Antibodies and isotype controls used in the flow cytometry analysis.

Marker	Description	Fluorochrome	Company	Cat. Number
**CD11b**	Integrin α M chain	FITC	Miltenyi Biotec	130-081-201
**CD11c**	Integrin α X subunit	PE/Cy5	BioLegend	301610
**CD14**	Lipopolysaccharide-binding protein	FITC	BioLegend	301804
**CD15**	Fucosyltransferase 4, SSEA-1	AF647	BioLegend	323012
**CD16**	Type III Fcγ receptor	PE/Cy7	Beckman Coulter	6607118
**CD27**	TNF-R superfamily, type I transmembrane glycoprotein	PerCP/Cy5.5	BioLegend	356408
**CD39**	NTPDase-1	PE	Miltenyi Biotec	130-093-503
**CD40**	TNF-receptor superfamily	FITC	BioLegend	334306
**CD44**	Extracellular matrix receptor-III	FITC	BioLegend	338804
**CD45**	Protein tyrosine phosphatase, receptor type C	FITC	BioLegend	304006
**CD45RA**	GP180	PE	BioLegend	304108
**CD49d**	Integrin α 4 chain	FITC	Beckman Coulter	IM1404U
**CD54**	ICAM-1	FITC	BioLegend	353108
**CD56**	Neural Cell Adhesion Molecule, NCAM	APC	Beckman Coulter	B10822
**CD62E**	ELAM-1	APC	Miltenyi Biotec	130-104-644
**CD63**	Type III lysosomal glycoprotein	PE	BioLegend	353004
**CD73**	5-nucleotidase, ecto	PE	BioLegend	344003
**CD80**	B7-1, B7, BB1	FITC	BioLegend	305206
**CD83**	BL11	APC	BD Biosciences	551073
**CD86**	B7-2, B70	PE	BioLegend	305406
**CD90**	Thy-1 membrane glycoprotein	PE/Cy7	BioLegend	328123
**CD95**	Fas, APO-1	PE/Cy7	BioLegend	305621
**CD114**	G-CSF Receptor	PE	BD Biosciences	554536
**CD116**	GM-CSF Receptor alpha	PE	BD Biosciences	551373
**CD117**	c-Kit	PE	Caltag Laboratories	LCD11704
**CD146**	Cell surface glycoprotein MUC18	APC	BioLegend	342011
**CD163**	Macrophage-associated antigen	APC/Cy7	BioLegend	333622
**CD184**	C-X-C Motif Chemokine Receptor 4	APC	BioLegend	306510
**CCR2**	CD192	AF647	BD Biosciences	558406
**CCR5**	CD195	APC	BD Biosciences	556903
**CCR6**	CD196	AF647	BioLegend	353403
**CCR7**	CD197	PE	BD Biosciences	552176
**CD209**	DC-SIGN	APC	BioLegend	330108
**CD274**	PD-L1	PE	BioLegend	329706
**CX3CR1**	C-X3-C Motif Chemokine Receptor 1	PE	BioLegend	355704
**Mouse IgG1**	κ Isotype Ctrl Antibody	FITC	BioLegend	400108
**Mouse IgG1**	κ Isotype Ctrl Antibody	PE	BioLegend	400112
**Mouse IgG1**	κ Isotype Ctrl Antibody	APC	BioLegend	400122
**Mouse IgG1**	κ Isotype Ctrl Antibody	AF647	BioLegend	400130
**Mouse IgG1**	κ Isotype Ctrl Antibody	APC/Cy7	BioLegend	400128
**Mouse IgG1**	κ Isotype Ctrl Antibody	PE/Cy5	BioLegend	400118
**Mouse IgG2a**	κ Isotype Ctrl Antibody	PE/Cy7	BioLegend	400232
**Mouse IgG2a**	κ Isotype Ctrl Antibody	PerCP/Cy5.5	BioLegend	400252

Supplementary Table 2. Primary antibodies used in western blot analysis.

Antibody	Company	Cat. Number
Rabbit polyclonal anti-Actin	Sigma-Aldrich	A2103
Rabbit polyclonal anti-Alix	Millipore	ABC40
Mouse monoclonal anti-Flotillin-1	BD Biosciences	610820
Mouse monoclonal anti-GAPDH	Santa Cruz Biotechnology	sc-32233
Rabbit polyclonal anti-GRP94	Cell Signaling Technology	2104
Mouse monoclonal anti-Lamin A/C	Cell Signaling Technology	4777
Rabbit polyclonal anti-TSG101	Proteintech	14497-1-AP
